# Development and Molecular Cytogenetic Identification of a New Wheat–*Psathyrostachys huashanica* Keng Translocation Line Resistant to Powdery Mildew

**DOI:** 10.3389/fpls.2021.689502

**Published:** 2021-06-07

**Authors:** Yuxiu Liu, Shuhua Huang, Jing Han, Chenchen Hou, Dasheng Zheng, Zhengmao Zhang, Jun Wu

**Affiliations:** ^1^College of Agronomy, Northwest A&F University, Yangling, China; ^2^College of Horticulture, Northwest A&F University, Yangling, China

**Keywords:** wheat, *P. huashanica*, translocation line, wheat powdery mildew, agronomic performance

## Abstract

*Psathyrostachys huashanica* Keng, a wild relative of common wheat with many desirable traits, is an invaluable source of genetic material for wheat improvement. Few wheat–*P. huashanica* translocation lines resistant to powdery mildew have been reported. In this study, a wheat–*P. huashanica* line, E24-3-1-6-2-1, was generated *via* distant hybridization, ethyl methanesulfonate (EMS) mutagenesis, and backcross breeding. A chromosome karyotype of 2*n* = 44 was observed at the mitotic stage in E24-3-1-6-2-1. Genomic *in situ* hybridization (GISH) analysis revealed four translocated chromosomes in E24-3-1-6-2-1, and *P. huashanica* chromosome-specific marker analysis showed that the alien chromosome fragment was from the *P. huashanica* 4Ns chromosome. Moreover, fluorescence *in situ* hybridization (FISH) analysis demonstrated that reciprocal translocation had occurred between the *P. huashanica* 4Ns chromosome and the wheat 3D chromosome; thus, E24-3-1-6-2-1 carried two translocations: T3DS·3DL-4NsL and T3DL-4NsS. Translocation also occurred between wheat chromosomes 2A and 4A. At the adult stage, E24-3-1-6-2-1 was highly resistant to powdery mildew, caused by prevalent pathotypes in China. Further, the spike length, numbers of fertile spikelets, kernels per spike, thousand-kernel weight, and grain yield of E24-3-1-6-2-1 were significantly higher than those of its wheat parent 7182 and addition line 24-6-3-1. Thus, this translocation line that is highly resistant to powdery mildew and has excellent agronomic traits can be used as a novel promising germplasm for breeding resistant and high-yielding cultivars.

## Introduction

Wheat (*Triticum aestivum* L.) is one of the most widely used agricultural crop species worldwide and serves as a staple food supply for at least one-third of the global population (Yang et al., 2016). Great progress has been made in wheat production through genetic improvement, breeding of locally adapted cultivars, and cultivation management (Fischer and Edmeades, 2010; Reynolds et al., 2012). However, wheat production is still limited by several factors such as diseases and relatively narrow genetic variation (Fischer and Edmeades, 2010; Mujeeb-Kazi et al., 2013). Powdery mildew, which is caused by *Blumeria graminis* (DC.) E.O. f. sp. *tritici* (*Bgt*), is one of the most destructive diseases constraining global wheat production (Dean et al., 2012; Morgounov et al., 2012). Fungicides are often used to control powdery mildew, but their widespread application has been hindered by high cost, the development of pathogen resistance, and environmental impacts (Khong et al., 2012). Breeding resistant cultivars is an effective and environmentally sound method to control powdery mildew (Tan et al., 2018). Unfortunately, owing to the presence of individual isolate-specific powdery mildew resistance genes (i.e., *Pm7* and *Pm17*), some resistant cultivars have become susceptible to pathogens (Rahmatov et al., 2016). Moreover, powdery mildew has become a widespread disease in major wheat production areas of China, resulting in severe reductions in yield and quality (He et al., 2015). Therefore, it is necessary to exploit new gene sources for resistance to powdery mildew and incorporate these genes into wheat. Moreover, by introducing genetic components of wild relatives into common wheat, distant hybridization is an effective method for producing new resistant germplasm and broadening genetic diversity (Lin et al., 2017).

*Psathyrostachys huashanica* Keng (2*n* = 2x = 14, NsNs), a wild relative of common wheat, is a Chinese endemic species that is found only in the Huashan Mountains of China (Kang et al., 2016). It has attracted substantial amounts of attention from wheat breeders due to its desirable traits, such as early maturity, increased numbers of kernels per spike, high tolerance to biotic stress, i.e., cold and drought, and high resistance to multiple diseases (Chen et al., 1991; Kang et al., 2008, 2016; Du et al., 2010, 2013a,b,c, 2014; Han et al., 2015, 2020; Li et al., 2019, 2020). To utilize the desirable traits of *P. huashanica*, distant crosses have been performed between *P. huashanica* and wheat since the 1980s (Chen et al., 1991). A series of wheat–*P. huashanica*-derived lines, including addition lines, substitution lines, translocation lines, and intergeneric amphiploids, have been developed and identified by molecular cytogenetic methods (Wang and Shang, 2000; Cao et al., 2008; Kang et al., 2008, 2016; Du et al., 2010, 2013a,b,c, 2014; Li et al., 2019, 2020; Bai et al., 2020). These derived lines with single *P. huashanica* chromosomes incorporated into the wheat genome exhibited better agronomic performance than their wheat parents, indicating that *P. huashanica* can be used as a valuable source of disease resistance and of several useful agronomic traits for wheat improvement.

In order to transfer alien genes, translocation lines are preferred by breeders (Falke et al., 2009) because of the smaller amount of alien genetic material, lower linkage drag, and regular meiotic behavior compared with wheat-alien species addition or substitution lines. Compared with its wheat parent, a small-segment wheat–*P. huashanica* translocation line presented more kernels per spike (Kang et al., 2016). Unfortunately, only a few wheat–*P. huashanica* translocation lines are available for wheat breeding. Moreover, they are poorly characterized (Cao et al., 2008; Wang et al., 2011; Ma et al., 2013; Kang et al., 2016). In addition, wheat–*P. huashanica*-derived lines resistant to powdery mildew have rarely been reported.

In this study, we developed a novel wheat–*P. huashanica* translocation line (E24-3-1-6-2-1) that is highly resistant to powdery mildew. The objectives were to (1) describe the development of the translocation line E24-3-1-6-2-1, (2) characterize the chromosome constitution of E24-3-1-6-2-1, (3) evaluate the powdery mildew resistance of E24-3-1-6-2-1, and (4) assess the agronomic performance of E24-3-1-6-2-1.

## Materials and Methods

### Development of the *P. huashanica* Translocation Line E24-3-1-6-2-1

*Psathyrostachys huashanica* (2*n* = 14, NsNs), winter wheat line 7182 (2*n* = 42, AABBDD), wheat–*P. huashanica* addition line 24-6-3-1, and wheat–*P. huashanica* translocation line E24-3-1-6-2-1 (2*n* = 44) were used in this study. The wheat–*P. huashanica* addition line 24-6-3-1 was harvested and its seeds were treated with ethyl methanesulfonate (EMS) at a dose of 1.0% and then planted in the field. Fresh pollen collected from the common wheat parent 7182 (2*n* = 42) was used to pollinated the spikes of M1 plants, which had been artificially emasculated 3–5 days prior. Mature hybrid seeds were harvested and used to produce a BC_1_F_1_ population. Pollen collected from the common wheat parent 7182 was used to pollinate the spikes of wheat–*P. huashanica* addition line 24-6-3-1 as a control. The plants with desirable agronomic traits and disease resistance were selected form the obtained plants, and then self-pollinated and simultaneously karyotyped *via* cytological examination and genomic *in situ* hybridization (GISH) analysis. The parental wheat line 7182 and *P. huashanica* were included as controls for evaluating powdery mildew resistance and agronomic performance and were used in expressed sequence tag (EST)-sequence-tagged site (STS) analyses. The common wheat cultivar Mingxian 169 has no known disease resistance genes and is susceptible to powdery mildew, so it was used as a susceptible control in powdery mildew response tests. Genomic DNA of Chinese Spring was used as a blocker in GISH analyses. These plant materials are preserved in the Shaanxi Key Laboratory of Genetic Engineering for Plant Breeding, College of Agronomy, Northwest A&F University, Shaanxi, China.

### Cytogenetic Analysis

The mitotic chromosomes of root tip cells (RTCs) of wheat–*P. huashanica* translocation line E24-3-1-6-2-1 were prepared and observed as previously described (Han et al., 2020). In brief, seeds of E24-3-1-6-2-1 were germinated in dishes. The root tips were cut, immersed in ice water for 24 h, and then transferred to an ethanol/acetic acid solution (3:1) for 1 week. After staining with 2% acetocarmine for at least 2 h, the root tips were squashed in 45% acetic acid and then subjected to subsequent cytological observations and GISH analysis. Cytological observations and documentation were performed using an Olympus BX60 microscope (Olympus BH2, Japan) equipped with a Photometrics SenSys charge-coupled device (CCD) camera (Penguin, Japan).

### GISH Analysis

Genomic *in situ* hybridization was performed to detect *P. huashanica* chromosomes in E24-3-1-6-2-1 according to a published method (Walling et al., 2005), with minor modifications (Han et al., 2020). The total genomic DNA was extracted from fresh leaves of *P. huashanica* and Chinese Spring according to the improved cetyl-trimethylammonium bromide (CTAB) method (Cota-Sánchez et al., 2006). Afterward, the DNA was labeled with Dig-Nick-Translation Mix/digoxigenin (digoxigenin-11-dUTP, DIG; Roche, Germany) using the nick translation method and then as hybridization probes for GISH. Chromosomes were counterstained with propidium iodide (PI), after which detection and visualization of the *P. huashanica* chromosomes were conducted according to Han et al. (2020).

### EST-STS Analysis

Expressed sequence tag-sequence-tagged site markers were used to determine the homoeologous relationships among the alien *P. huashanica* chromosomes. The total genomic DNA was extracted from the translocation line E24-3-1-6-2-1 and parents following the methods of Cota-Sánchez et al. (2006). A total of 83 EST-STS multiple-locus primer pairs^1^ were used to identify the *P. huashanica* chromosome in E24-3-1-6-2-1; the primers were distributed evenly among seven wheat homoeologous groups. PCR-based amplification of EST-STS markers was performed, and the products were separated and visualized as previously described (Han et al., 2020).

### FISH Analysis

Fluorescence *in situ* hybridization (FISH) was conducted using the oligonucleotide probes Oligo-pTa535-1 (Tamra-5'AAAAACTTGA CGCACGTCAC GTACAAATTG GACAAACTCT TTCGGAGTAT CAGGGTTC, red) and Oligo-pSc119.2 (6-FAM-5'CGTTTTGTG GACTATTACT CACCGCTTTG GGGTCCCATA GCTAT, green) according to Patokar et al. (2016) and Lang et al. (2019) after rinsing the GISH probe signals. The Oligo-pTa535-1 probe was used to identify the A and D genomes of hexaploid wheat, while the Oligo-pSc119.2 probe was used to identify the B genome (Tang et al., 2014; Kang et al., 2016; Lang et al., 2019). Observations and photomicrographs of chromosomes were conducted and collected, respectively, using an Olympus BX60 microscope (Olympus BH2, Japan) equipped with a Photometrics SenSys CCD camera (Penguin, Japan).

### Evaluation of Powdery Mildew Response

Responses to powdery mildew were determined for E24-3-1-6-2-1, its parents and the susceptible cultivar Mingxian 169 at the adult stage using three replicates during the 2018–2019 and 2019–2020 cropping seasons at the Yangling Wheat Experimental Station, Northwest A&F University, Yangling, Shaanxi, China (34°16'56.24"N, 108°4'27.95"W). The powdery mildew response recorded in rows was separated from those for the assessment of agronomic traits. Artificial inoculations were conducted at the jointing stage by applying a mixture of *Bgt* isolates that are prevalent in the major wheat-producing areas of China (Zhou et al., 2002) evenly over the leaves until the susceptible check was fully infected. When the susceptible control (Mingxian 169) showed fully developed conidia, the reactions were evaluated and recorded on a 0–9 rating scale, where 0–4 indicated resistance and 5–9 indicated susceptibility, in accordance with the methods of Sheng and Duan (1991).

### Assessment of Agronomic Traits

Translocation line E24-3-1-6-2-1, addition line 24-6-3-1, and their parents 7182 and *P. huashanica* were planted in 9.0 m × 1.2 m plots, with six rows per plot and 0.20 m between rows. The field experimental plots were arranged following a randomized complete block design (with three replications) in Yangling (34°18'14"N, 108°5'38"W) during the 2018–2019 and 2019–2020 cropping seasons. When they reached maturity (Feekes 11.3–11.4; Miller, 1999), all plots were harvested using a small-plot combine [4LZ-2.5 (PR0688Q), Kubota Agricultural Machinery (Suzhou) Co., Ltd.] to evaluate yield-related traits, including plant height, spike length, number of spikes per square meter, number of spikelets per spike, number of kernels per spike, thousand-kernel weight, and grain yield. Duncan’s multiple range test, which was conducted using the general linear model procedure in SAS package (version 9, SAS Institute Inc., Cary, NC, United States), was used to test for significant differences between E24-3-1-6-2-1, 24-6-3-1, and its parents for all the measured traits.

## Results

### Development of the *P. huashanica* Translocation Line E24-3-1-6-2-1

**Figure 1:**
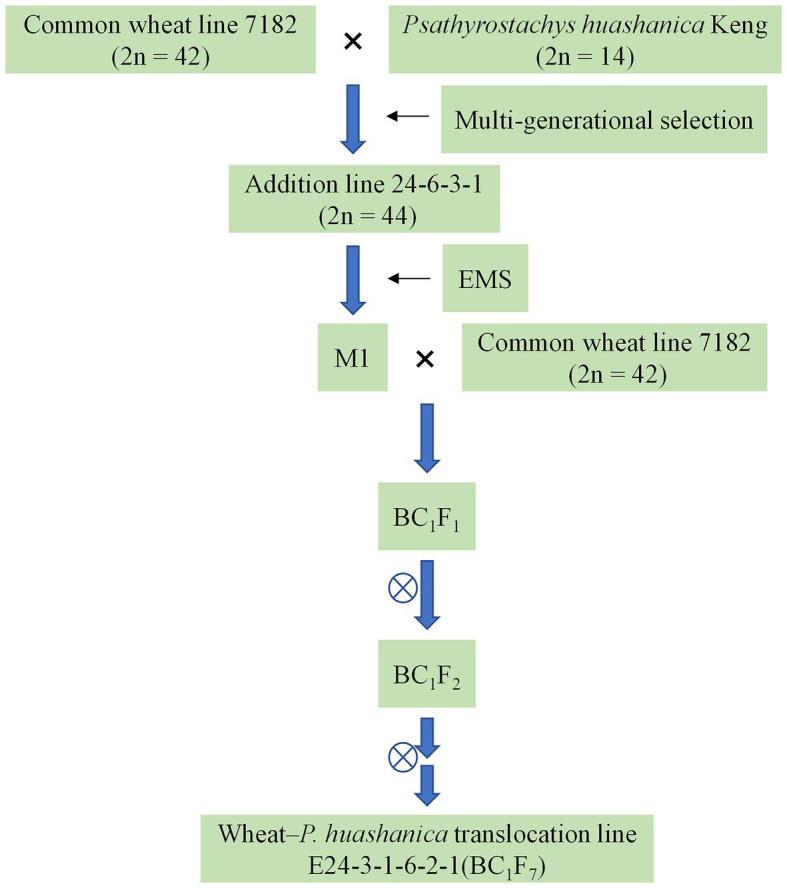
Scheme of the development of wheat–*Psathyrostachys huashanica* translocation line E24-3-1-6-2-1.

**Figure 2:**
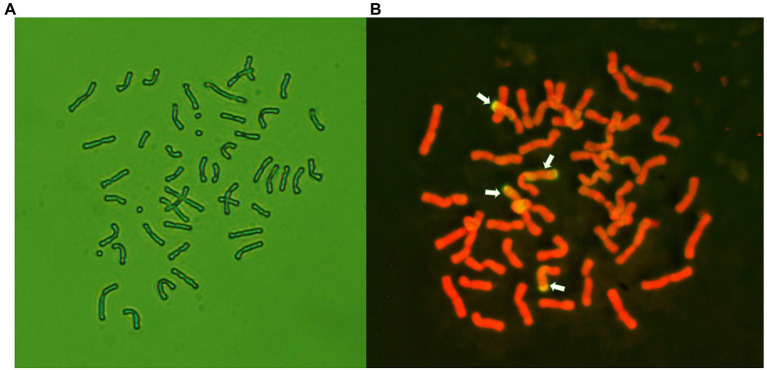
Cytological and genomic *in situ* hybridization (GISH) analysis of line E24-3-1-6-2-1. **(A)** Mitotic metaphase, 2*n* = 44. **(B)** GISH analysis of the chromosome constitution of E24-3-1-6-2-1. GISH was conducted using *Psathyrostachy shuashanica* DNA as a probe and Chinese Spring DNA as a blocker. Four chromosomes with fluorescent hybridization signals (yellow-green) were identified as having alien segments in E24-3-1-6-2-1. The chromosomes were counterstained with propidium iodide (PI; red).

Distant hybridization between the winter wheat line 7182 and *P. huashanica* was performed in 1991. The wheat–*P. huashanica* addition line 24-6-3-1 was produced *via* multigenerational selection; this line has a chromosome number of 2*n* = 44, a large number of tillers (Du et al., 2014), and good visual grain quality. Seeds of addition line 24-6-3-1 were treated by 1.0% EMS. The M1 plants were backcrossed with the wheat parent 7182. The BC_1_F_2_ population (50 lines) was then advanced to the BC_1_F_6_ generation by single-seed descent (Figure 1). From the BC_1_F_1_ to BC_1_F_6_ generations, the selfed progeny of plants were tested for resistance to a mixture of *Bgt* isolates that are prevalent in the major wheat-producing areas of China. Plants with desirable agronomic traits and disease resistance were selected and self-pollinated by covering the spikes with white paper bags, and the plants were simultaneously karyotyped *via* cytological examination and GISH analysis. One of the isolated translocation lines, E24-3-1-6-2-1 (BC_1_F_7_), whose chromosome number was 2*n* = 44 (Figure 2A), was homozygous and was subsequently maintained by self-pollination.

### GISH Analysis of E24-3-1-6-2-1

Using total genomic DNA from *P. huashanica* as a probe and that from Chinese Spring as the blocker, GISH analysis, conducted on mitotic metaphase cells to determine the chromosome configuration of line E24-3-1-6-2-1, demonstrated that E24-3-1-6-2-1 had four chromosome segments with yellow-green hybridization signals and 40 chromosomes with red signals caused by counterstaining with propidium iodide (PI). Two of the signals were emitted from nearly 2/3 of the wheat chromosome, obviously covering the long arm and partial short arm of the chromosome connected by the centromere, while two of the signals were emitted from nearly half of the wheat chromosome, clearly covering the short arm (Figure 2B); these results suggested that chromosome segments of *P. huashanica* had been translocated into the wheat chromosome. Therefore, E24-3-1-6-2-1 was confirmed to have a large segmental translocation wheat–*P. huashanica*.

### EST-STS Analysis of E24-3-1-6-2-1

**Figure 3:**
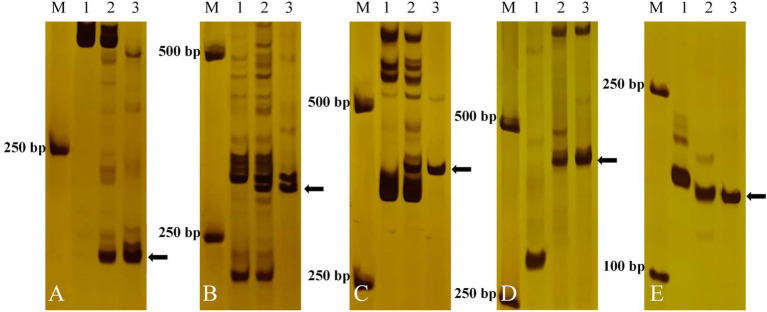
Expressed sequence tag (EST)-sequence-tagged site (STS) marker analysis of E24-3-1-6-2-1 and its parents. Five pairs of EST-STS markers [**(A)** BE442811, **(B)** BE44606, **(C)** BQ161513, **(D)** BF473854, and **(E)** CD373484] corresponding to homoeologous Group 4 amplified the Ns chromosome-specific bands in the E24-3-1-6-2-1 line and *P. huashanica*. Lane M: DL2000 marker; Lane 1: line 7182; Lane 2: line E24-3-1-6-2-1; and Lane 3: *P. huashanica*. Diagnostic amplification products of the Ns genome are indicated by arrows.

To determine the homoeologous groups of the translocated wheat chromosome in E24-3-1-6-2-1, 89 pairs of EST-STS markers from seven homoeologous groups of common wheat were selected to screen for polymorphisms in E24-3-1-6-2-1 as well as its parents 7182 and *P. huashanica*. Specific bands were obtained with five pairs of EST-STS primers, namely, BE442811, BE446061, BQ161513, BF473854, and CD373484, from homoeologous group-4 chromosomes (4AL, 4AS, 4BL, 4BS, 4DL, and 4DS). In addition, they were different from the bands that were amplified in common wheat 7182 (Figure 3), indicating that the five EST-STS markers were Ns genome-specific and that the alien chromosome segment in E24-3-1-6-2-1 was from *P. huashanica* 4Ns.

### FISH Analysis of E24-3-1-6-2-1

**Figure 4:**
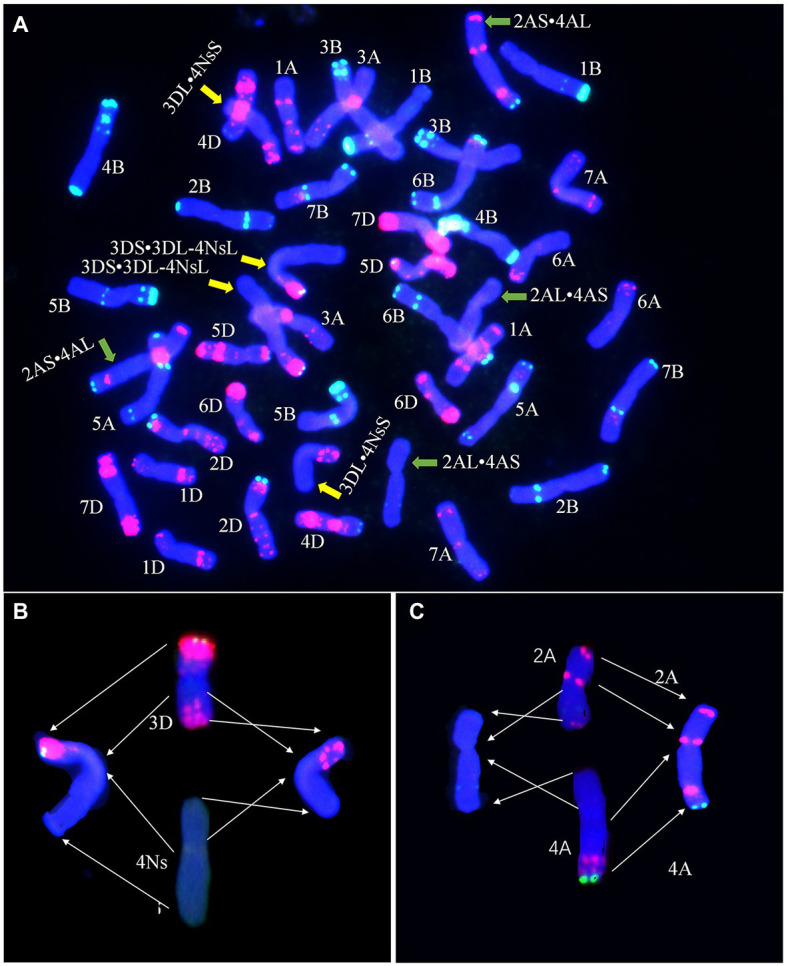
Fluorescence *in situ* hybridization (FISH) analysis of E24-3-1-6-2-1 **(A)**. Oligo-primers pSc119.2 (green) and pTa535-1 (red) were used as probes for wheat chromosomes. The wheat 3D chromosome and *P. huashanica* 4Ns chromosome, as well as the wheat 2A and 4A chromosomes underwent translocations to form 3DS·3DL-4NsL, 3DL·4NsS, 2AL·4AS, and 2AS·4AL chromosomes, as indicated by the arrows. Diagrams of translocations [3DS·3DL-4NsL and 3DL·4NsS **(B)**, and 2AL·4AS and 2AS·4AL **(C)**] in E24-3-1-6-2-1 showing breakage sites in each chromosome, as indicated by the arrows, and chromosome rearrangements. The chromosomes were stained with 4',6-diamidino-2-phenylindole (DAPI; blue).

Fluorescence *in situ* hybridization analysis with probes Oligo-pTa535 and Oligo-pSc119.2 was used to identify the translocated wheat chromosome in line E24-3-1-6-2-1. The FISH results showed that the translocation happened on chromosome 3D (Figure 4A). The breakage site was near the centromere of 3DL and was caused by the loss of most of chromosome 3D (3DS·3DL). The chromosome 3D (3DS·3DL) segment was combined with the newly acquired 4Ns chromosome segment (4NsL) of *P. huashanica* to form 3DS·3DL-4NsL, whereas the remaining 3DL segment and the 4Ns chromosome segment (4NsS) of *P. huashanica* formed a second translocation chromosome, 3DL·4NsS (Figure 4B). A Robertsonian translocation also occurred between wheat chromosomes 2A and 4A, forming two new chromosomes, 2AL·4AS and 2AS·4AL (Figure 4C). Thus, E24-3-1-6-2-1 was confirmed to have two translocations.

### Responses of E24-3-1-6-2-1 to Powdery Mildew

**Figure 5:**
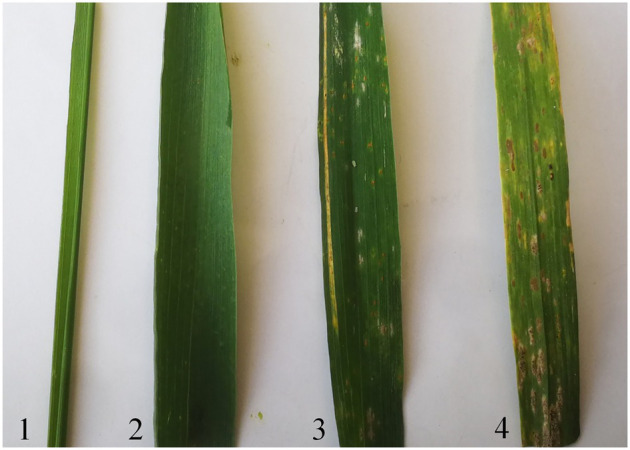
Powdery mildew responses of *P. huashanica*, line E24-3-1-6-2-1, line 7182, and Mingxian 169 (from 1 to 4) to a mixture of 30 powdery mildew (*Bgt*) isolates at the adult stage.

In two consecutive wheat growing seasons (2018–2020), translocation line E24-3-1-6-2-1, 7182, *P. huashanica*, and Mingxian 169 were assessed at the adult stage to determine their response to powdery mildew in the field. The results showed that E24-3-1-6-2-1 was highly resistant to powdery mildew (infection type 1), while *P. huashanica* was immune (infection type 0). In contrast, 7182 and Mingxian 169 were susceptible with infection types 5 and 8, respectively (Figure 5).

### Agronomic Performance of E24-3-1-6-2-1

**Figure 6:**
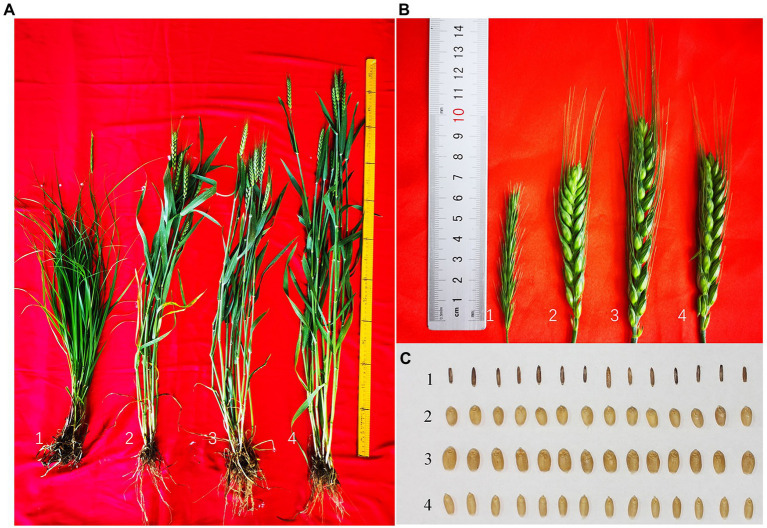
Plant morphology of wheat–*P. huashanica* translocation line E24-3-1-6-2-1 and its parents. **(A)** Adult plant, **(B)** spikes, and **(C)** kernels. Numbers 1–4 represent *P. huashanica*, 7182, line E24-3-1-6-2-1, and line 24-6-3-1, respectively.

E24-3-1-6-2-1 was significantly taller than *P. huashanica* and 7182 but significantly shorter than addition line 24-6-3-1, while the significantly longest spike length was found for E24-3-1-6-2-1 in the two cropping seasons (*p* < 0.05; Figures 6A,B; Table 1). There were no significant differences in spike number between E24-3-1-6-2-1 and line 7182, but the highly significant increase in spike number was found for addition line 24-6-3-1 (*p* < 0.05), compared with 7182 and translocation lines, suggesting that 24-6-3-1 resembled the female parent *P. huashanica* and tillered profusely (Table 1). E24-3-1-6-2-1 had significantly higher numbers of fertile spikelets per spike and numbers of kernels per spike than lines 7182 and 24-6-3-1 in the two seasons (*p* < 0.05; Figure 6B; Table 1). The significantly highest thousand-kernel weight and grain yield were observed for E24-3-1-6-2-1 among all the lines (*p* < 0.05; Figure 6C; Table 1).

**Table 1:** Agronomic performance of the wheat–*P. huashanica* translocation line E24-3-1-6-2-1, its parents, and addition line 24-6-3-1.

Season	Line	Plant height (cm)	Spike length (cm)	Number of spikes per m^2^	Number of fertile spikelets per spike	Number of kernels per spike	Thousand-kernel weight (g)	Grain yield (t/ha)
2018–2019	*P. huashanica*	50.8 ± 4.25d	6.6 ± 0.55c	Clump	15.7 ± 1.53b	-	4.1 ± 0.35d	-
	7182	62.7 ± 2.40c	7.3 ± 0.15c	508.7 ± 10.69b	13.0 ± 1.00c	25.3 ± 0.75c	37.2 ± 0.60b	4.17 ± 0.21c
	E24-3-1-6-2-1	70.7 ± 2.52b	9.9 ± 0.53a	525.0 ± 7.00b	18.0 ± 1.00a	34.4 ± 0.74a	41.5 ± 0.82a	5.32 ± 0.13a
	24-6-3-1	102.0 ± 2.65a	8.3 ± 0.20b	588.0 ± 7.00a	15.3 ± 0.58b	30.9 ± 0.70b	30.3 ± 0.78c	4.68 ± 0.17b
2019–2020	*P. huashanica*	51.8 ± 3.25d	6.4 ± 0.60d	Clump	16.3 ± 1.15b	-	4.3 ± 0.45d	-
	7182	61.3 ± 1.53c	7.5 ± 0.15c	476.0 ± 7.00c	14.0 ± 1.00c	26.3 ± 0.87c	38.3 ± 0.80b	4.32 ± 0.18c
	E24-3-1-6-2-1	72.0 ± 3.00b	10.3 ± 0.20a	508.7 ± 5.35b	19.0 ± 1.00a	36.5 ± 0.93a	42.3 ± 1.45a	5.59 ± 0.12a
	24-6-3-1	103.7 ± 1.53a	8.7 ± 0.20b	561.2 ± 8.81a	16.7 ± 0.58b	33.1 ± 1.53b	32.2 ± 1.25c	5.03 ± 0.27b

## Discussion

Wheat is a self-pollinating plant species, and long-term breeding has narrowed its genetic diversity and may lead to the loss of many useful genes for stress resistance and adaptation. Moreover, the limited genetic diversity has hindered its yield improvement in recent years (Mujeeb-Kazi et al., 2013). Broadening the genetic base is considered an important way to improve disease resistance and agronomic traits of wheat (Tester and Langridge, 2010). Chromosome engineering is a desirable method for not only broadening wheat diversity but also effectively transferring elite traits from alien species into common wheat to improve productivity (Jiang et al., 1994; Friebe et al., 1996; Qi et al., 2007; Zhao et al., 2010). Developing wheat-alien species translocation lines and determining their chromosome constitutions are crucial steps to the introgression of elite genes to wheat (Jiang et al., 1994; Gill et al., 2011; Kang et al., 2016). Conventional chromosomal manipulation by crossing wheat and distant hybridization by crossing alien species and wheat have been used to induce chromosome translocations (Jiang et al., 1994; Friebe et al., 1996). Hybridization between common wheat and alien species including *Haynaldia villosa* (L.) Schur [syn. *Dasypyrum villosum* (L.) P. Candargy], *Agropyron cristatum* (L.), *P. huashanica*, and *Thinopyrum intermedium* has been conducted to generate many translocation lines in wheat breeding programs (Chen et al., 2013; Ye et al., 2015; Zhan et al., 2015; Zhang et al., 2015; Patokar et al., 2016; Han et al., 2020; Li et al., 2020). One strategy, which involved the use of ^60^Co γ-radiation, the Chinese Spring ph1b mutant, gametocidal chromosomes originating from *Aegilops*, and backcrossing, was employed to produce the small-segment translocation line WR35 (An et al., 2019). Some translocation lines, such as the wheat-rye 1BL·1RS translocation line, have been identified through *in situ* hybridization and molecular identification techniques; these lines are considered the most successful examples of disease resistance improvement in wheat by chromosome engineering (Ren et al., 2012; Howell et al., 2014). Some translocation lines were induced from wheat–*A. cristatum* 2P disomic addition line II-9-3 with highly resistant to powdery mildew and leaf rust by ^60^Co-γ irradiation and gametocidal chromosome 2C (Li et al., 2016). Of chemical mutagens, EMS mutagenesis in plant is the most widely used mutagenesis technique, which causes random point mutations by selectively alkylating guanine to cause base conversion or substitution (Sikora et al., 2011). Chromosome breakage in *Drosophila melanogaster* and *Vicia faba* were induced by EMS (Natarajan and Upadhya, 1964; Bishop and Lee, 1974). A and B chromosome translocations were observed in the pearl millet carrying B-chromosome, which was induced by EMS treatment (Pushpa, 1980). Sixty-one wheat–*P. huashanica* translocation lines were induced from wheat–*P. huashanica* disomic addition line by 0.8–1.2% (v/v) EMS and the translocation frequency was 6.56% *via* cytological observation and GISH analysis (Jing et al., 2015). About 1.0% (v/v) EMS was the optional concentration for inducing wheat–*P. huashanica* translocation lines (Jing et al., 2015). In the present study, a new wheat–*P. huashanica* translocation line (E24-3-1-6-2-1) was developed through distant hybridization, EMS mutagenesis, and backcrossing with common wheat; this line was characterized by a combined analysis including GISH, FISH, and *P. huashanica* chromosome-specific markers, as well as an assessments of powdery mildew resistance and agronomic performance.

Alien genetic resources are important in breeding program for increasing yield and quality and for improving the stress resistance of wheat. Different types of derived lines have been generated by the genetically distant cross between *P. huashanica* and common wheat 7182. However, these derived lines can be adopted by breeders for wheat improvement only after they have been accurately identified. Indeed, methods are available for identifying alien chromosomes and their segments in the derived lines. For instance, cytological observations and identification by *in situ* hybridization (i.e., GISH and FISH) were performed in the present study to detect the presence of alien chromosomes in the derived lines. In particular, FISH is a powerful and accurate tool for distinguishing all 21 common wheat chromosomes pairs in mitotic cells and determining the size and breakpoint positions of the chromosomes when synthetic oligonucleotides are used as probes (Tang et al., 2014). Molecular marker analysis based on PCR is an essential technique for determining the homoeology of alien chromosomes. In the present study, the progeny of E24-3-1-6-2-1 derived from three consecutive selfed generations were confirmed to be genetically stable *via* GISH analysis. In addition, GISH indicated that E24-3-1-6-2-1 was a wheat–*P. huashanica* large-segment translocation line (Figure 2B). Five of 83 EST-STS pair markers specific to 4Ns of *P. huashanica* indicate that alien chromosomes of E24-3-1-6-2-1 were from the 4Ns chromosome of *P. huashanica* (Figure 3). The oligonucleotide probes Oligo-pTa535-1 and Oligo-pSc119.2 were used in FISH analysis to precisely determine that E24-3-1-6-2-1 was a wheat–*P. huashanica* 3DS·3DL-4NsL and 3DL·4NsS translocation line (Figures 4A–C).

The high variability of pathogens and uniformity of resistance sources has resulted in the rapid loss of powdery mildew resistance, despite powdery mildew severely hindering the grain yield and quality improvement of wheat (Cowger et al., 2012; Yang et al., 2016; He et al., 2017; Xu et al., 2018). Thus, it is urgent that new powdery mildew resistance gene sources can be identified and used to develop new resistant germplasms. The wheat–*P. huashanica* addition line H5-5-4-2 was highly resistant to powdery mildew at both the adult and seedling stages (Han et al., 2020). A genetically stable wheat–*P. huashanica* T3DS-5NsL·5NsS and T5DL-3DS·3DL translocation line was more resistant to powdery mildew than its wheat parents at both the adult and seedling stages (Li et al., 2020). Unfortunately, few wheat–*P. huashanica* progeny lines that are completely resistant or highly resistant to powdery mildew have been identified. The addition line 24-6-3-1 was highly resistant to powdery mildew at the adult stages (data not shown). In the present study, the translocation line E24-3-1-6-2-1 at the adult stage was highly resistant to a mixture of *Bgt* isolates, the findings of which are similar to those for its *P. huashanica* parent, while its wheat parent 7182 was susceptible to powdery mildew (Figure 5), suggesting that the powdery mildew resistance of E24-3-1-6-2-1 was from *P. huashanica*. These results also indicated that the chromosome segment with the resistance gene of *P. huashanica* was successfully transferred into the 7182 background. Previously, wheat–*P. huashanica* lines with *P. huashanica* chromosome 4Ns had not been identified as being resistant to powdery mildew. These findings provide strong support for exploring resistance-associated loci in *P. huashanica* and developing novel resistant germplasms.

Compensations between yield components are often employed to improve wheat yield (Slafer et al., 2014). The primary components of grain yield are the number of spikes per hectare, number of kernels per spike, and thousand-kernel weight. Numbers of spikelets per spike and number of kernels per spike have been the most important parameters among the many potential traits that determine wheat yield during the long-term breeding process (Zhou et al., 2007). Zhang et al. (2015) identified a *T. aestivum*–*D. villosum* translocation line with increased spike length, increased spikelet number, and increased grains per spike. Similarly, a wheat–*P. huashanica* translocation line with elongated spikes and increased kernel number per spike has been reported (Li et al., 2020). The presence of chromosome 4Ns from *P. huashanica* in the wheat 7182 background resulted in significantly increased tiller number, increased kernel number per spike, and increased spike length (Du et al., 2014). In the present study, compared with its wheat parent 7182 and addition line 24-6-3-1, E24-3-1-6-2-1 also presented greater spike length, number of fertile spikelets, kernel number per spike, thousand-kernel weight, and grain yield (Figures 6A,B; Table 1). The increased values of the yield components strongly reflect the significantly increased grain yield of E24-3-1-6-2-1 (Table 1). Therefore, translocation line E24-3-1-6-2-1, which has a *P. huashanica* fragment with excellent agronomic traits (Table 1) and is highly resistant to powdery mildew (Figure 5), can be used as a donor to provide genes for the genetic improvement of wheat. The genes that provide powdery mildew resistance in E24-3-1-6-2-1 are currently being identified from the 4NsS or 4NsL chromosome arms of *P. huashanica* by using 4N-specific markers and *via* GISH and FISH approaches. Additional genetic and molecular mapping studies are needed to further identify the powdery mildew resistance in E24-3-1-6-2-1.

In fact, for wheat breeding, breeder needs individuals with 42 chromosomes that contain the wheat *P. huashanica* chromosomes. In response to it, the offspring of the translocation line E24-3-1-6-2-1 and the common wheat parents 7182 have been obtained. Many progenies were also obtained by offspring lines selfing. Progenies are being identified by cytological observation and GISH analysis to find individuals required with desirable agronomic traits. At the same time, specific molecular markers for translocation fragment are being developed for more convenient and effective selection.

## Conclusion

We studied the development, chromosomal constitution, powdery mildew response, and agronomic performance of wheat–*P. huashanica* translocation line E24-3-1-6-2-1. This line was identified as a new wheat–*P. huashanica* T3DS·3DL-4NsL and T3DL·4NsS translocation line that contains the *P. huashanica* chromosome segments that confer powdery mildew resistance and increased spike length, number of fertile spikelets, kernel number per spike, and thousand-kernel weight in wheat. E24-3-1-6-2-1 is not only a potential powdery mildew-resistant germplasm but also an intermediate material for breeding high-yielding wheat.

## Data Availability Statement

The original contributions presented in the study are included in the article/supplementary material, further inquiries can be directed to the corresponding author.

## Material Availability Statement

The translocation line E24-3-1-6-2-1 used in this study is available from the corresponding author to be required for academic study.

## Author Contributions

YL and JW conceived and designed the study. YL, SH, JH, CH, DZ, and JW performed the experiments. YL, SH, ZZ, and JW analyzed the data, prepared the figures and/or tables, and wrote the paper. All authors contributed to the article and approved the submitted version.

### Conflict of Interest

The authors declare that the research was conducted in the absence of any commercial or financial relationships that could be construed as a potential conflict of interest.

## Footnotes

^1^https://wheat.pw.usda.gov/SNP/new/pcr_primers.shtml
